# Investigating the Physical Effects in Bacterial Therapies for Avascular Tumors

**DOI:** 10.3389/fmicb.2020.01083

**Published:** 2020-06-04

**Authors:** Pietro Mascheroni, Michael Meyer-Hermann, Haralampos Hatzikirou

**Affiliations:** ^1^Braunschweig Integrated Centre of Systems Biology and Helmholtz Centre for Infection Research, Braunschweig, Germany; ^2^Centre for Individualized Infection Medicine, Hannover, Germany; ^3^Institute for Biochemistry, Biotechnology and Bioinformatics, Technische Universität Braunschweig, Braunschweig, Germany

**Keywords:** cancer, bacterial therapy, mathematical modeling, chemotaxis, space competition

## Abstract

Tumor-targeting bacteria elicit anticancer effects by infiltrating hypoxic regions, releasing toxic agents and inducing immune responses. Although current research has largely focused on the influence of chemical and immunological aspects on the mechanisms of bacterial therapy, the impact of physical effects is still elusive. Here, we propose a mathematical model for the anti-tumor activity of bacteria in avascular tumors that takes into account the relevant chemo-mechanical effects. We consider a time-dependent administration of bacteria and analyze the impact of bacterial chemotaxis and killing rate. We show that active bacterial migration toward tumor hypoxic regions provides optimal infiltration and that high killing rates combined with high chemotactic values provide the smallest tumor volumes at the end of the treatment. We highlight the emergence of steady states in which a small population of bacteria is able to constrain tumor growth. Finally, we show that bacteria treatment works best in the case of tumors with high cellular proliferation and low oxygen consumption.

## 1. Introduction

Cancers display huge variability between different patients and even in the same patient. Nonetheless, cancer cells share a finite set of hallmarks such as sustained proliferation, invasion and metabolic reprogramming, which shape their behavior in solid tumors (Hanahan and Weinberg, 2011). Among other hallmarks, tumor cells are known to recruit new blood vessels to sustain their proliferation, in a process known as *tumor angiogenesis* (Folkman, 1971). This neovasculature is generally altered in terms of architecture and morphology of the vessels, leading to poor perfusion of certain areas of the tumor (Carmeliet and Jain, 2000). Hypoxic regions are thus created and maintained during tumor development, concurring to the progression of cancer cells toward malignant phenotypes (Vaupel and Mayer, 2007). Moreover, low nutrient levels can lead to cell quiescence, a situation in which tumor cells delay metabolic activities and become less sensitive to standard chemotherapies (Challapalli et al., 2017). Such hypo-perfused areas are generally associated with poor patient outcome but, on the other hand, could be exploited for tumor targeting (Wilson and Hay, 2011). The same hypoxic areas provide indeed a niche for bacteria to colonize the tumor and exert a therapeutic action (Forbes, 2010; Zhou et al., 2018). The use of bacteria for cancer therapy dates back hundreds of years, with doctors reporting tumor regression in several patients (Kramer et al., 2018). However, such treatments also caused some fatalities and the limited understanding of the therapeutic mechanisms of action shifted research efforts toward other strategies - especially radiotherapy (Kramer et al., 2018). In the last few years the use of live bacteria for cancer treatment has regained interest, and several bacterial strains have been tested in animal models and even advanced to clinical trials (Torres et al., 2018). Nevertheless, clinical development of such therapies is still facing significant issues due to infection-associated toxicities and incomplete knowledge of infection dynamics (Kramer et al., 2018; Zhou et al., 2018). As much research was focused on the immune responses after bacteria administration, a clear picture of the interaction between cancer and bacterial cells is still lacking.

Mathematical modeling emerges as a promising candidate to assist the understanding of bacterial therapy mechanism of action in cancer. Mathematical models have been applied in the context of cancer to elucidate its progression and treatment (Byrne, 2010; Altrock et al., 2015). Recent examples combining experimental and modeling work in bacterial therapies are given in Kasinskas and Forbes (2006), Jean et al. (2014), Hatzikirou et al. (2017), and Suh et al. (2018), featuring *in vitro* as well *in vivo* experiments.

Here we describe a mathematical model for bacteria-based cancer therapy within avascular tumors, focusing on the influence of physical effects on therapy outcomes. Such effects are present in every biological system but are often concealed by the complexity of the interactions between molecular and cellular players. Here, we show through a simple mathematical model that these effects take an important part in bacterial therapies and are able to influence their outcomes. The model is formulated in the context of mixture theory, a framework with a long history of applications to biological problems—see for example Ambrosi and Preziosi (2002); Breward et al. (2001, 2002, 2003); Byrne and Preziosi (2003); Chaplain et al. (2006); Preziosi and Tosin (2009) and the recent reviews of Siddique et al. (2017) and Pesavento et al. (2017). Our aim is to evaluate the impact of bacterial chemotaxis and anti-tumor activity on cancer cells, using spheroids as a prototype of avascular tumors. We consider bacterial administration after full formation of the spheroid, when hypoxic areas are present. We describe the effects of the treatment on the behavior of the spheroid constituents, e.g., tumor cells and bacteria volume fractions, at different time points and over the spheroid radius.

The remainder of the paper is organized as follows. In section 2, we describe the mathematical model and its derivation. In section 3 we present model results, analyzing the impact of different model parameters. Finally, in section 4 we discuss the biological implications of the results and suggest new research directions.

## 2. Materials and Methods

We propose a mathematical model describing the impact of bacterial cells on tumor spheroid growth. The model is based on mixture theory, a continuum theory that allows to describe the chemo-mechanical interactions between different tissue components. We follow the approach discussed in Preziosi (2003) and Byrne (2012) and, specifically, adapt the derivation in Boemo and Byrne (2019) to our problem. In the following we present the final form of the equations, leaving the full derivation in the Supplementary Information.

We describe the tumor as being composed of three main constituents (or *phases* in the language of mixture theory): tumor cells (TCs), bacteria and extracellular material. The variables referring to these quantities will be identified by the indexes c, b, and f, respectively. We also consider the presence of a nutrient, i.e., oxygen, diffusing over the spheroid domain. The model equations are derived by applying conservation of mass and linear momentum to each phase, and enforcing the saturation constraint (i.e., all the space in the spheroid is occupied by the phases, there are no voids). Then, we impose suitable assumptions regarding the mechanical properties of the model constituents and their interaction terms.

### 2.1. Model Equations

In the following we will be interested in the case of tumor spheroids, for which the assumption of spherical symmetry applies. The problem reduces to the set of Partial Differential Equations (PDEs):

(1) $$\begin{array}{rcl} \frac{\partial \phi_{\text{c}}}{\partial t} & = & \frac{1}{r^{2}}\frac{\partial }{\partial r}\left\{r^{2}\left[D_{\text{c}}\left(1-\phi_{\text{c}}\right)\frac{\partial \phi_{\text{c}}}{\partial r}-D_{\text{b}}\phi_{\text{c}}\frac{\partial \phi_{\text{b}}}{\partial r}-\chi \phi_{\text{c}}\phi_{\text{b}}\frac{\partial n}{\partial r}\right]\right\}\\ + & S_{\text{c}}, \end{array}$$

(2) $$\begin{array}{l} \frac{\partial \phi_{\text{b}}}{\partial t}=\frac{1}{r^{2}}\frac{\partial }{\partial r}\{r^{2}[D_{\text{b}}\left(1-\phi_{\text{b}}\right)\frac{\partial \phi_{\text{b}}}{\partial r}-D_{\text{c}}\phi_{\text{b}}\frac{\partial \phi_{\text{c}}}{\partial r}\\ \;+\chi \phi_{\text{b}}\left(1-\phi_{\text{b}}\right)\frac{\partial n}{\partial r}]\}+S_{\text{b}}, \end{array}$$

(3) $$\begin{array}{rcl} \frac{\partial n}{\partial t} & = & \frac{1}{r^{2}}\frac{\partial }{\partial r}\left(r^{2}D_{\text{n}}\frac{\partial n}{\partial r}\right)+S_{\text{n}}. \end{array}$$

Here, ϕ_c_, ϕ_c_, and *n* are the tumor cell and bacteria volume fractions and normalized nutrient concentration, respectively. These quantities depend on the radial coordinate *r* ∈ [0, *R*] and time *t* ∈ [0, *t*_*f*_]. In addition, *D*_i_ are the phases motility coefficients (i = c,b), *D*_n_ the nutrient diffusion coefficient, and χ the bacterial chemotactic coefficient. The mass exchange terms *S*_i_ (i = c,b,n), regulating the transfer of mass between the different components, will be detailed in the next subsection. Note that we do not solve explicitly for ϕ_f_ (i.e., the volume fraction of extracellular material) since this quantity can be obtained as ϕ_f_ = 1−ϕ_c_−ϕ_b_ due to the saturation constraint (see the Supplementary Information). We model growth of the spheroid as a free-boundary problem, in which the outer tumor radius *r* = *R*(*t*) moves with the same velocity as the TC phase,

(4) $$\begin{array}{r} \frac{dR}{dt}=v_{\text{c}}\left(R,t\right)=D_{\text{b}}\frac{\partial \phi_{\text{b}}}{\partial r}+D_{\text{c}}\left(1-\frac{1}{\phi_{\text{c}}}\right)\frac{\partial \phi_{\text{c}}}{\partial r}+\chi \phi_{\text{b}}\frac{\partial n}{\partial r}{|}_{r=R}. \end{array}$$

Finally, we define a set of boundary and initial conditions to close the differential problem in Equations (1)–(3). Due to the problem symmetry no-flow boundary conditions are enforced at the spheroid center, whereas we fix the values of TC volume fraction, bacterial volume fraction and normalized nutrient concentration on the spheroid boundary:

(5) $$\begin{array}{r} \partial_{r}\phi_{\text{c}}=\partial_{r}\phi_{\text{b}}=\partial_{r}n=0,\;r=0 \end{array}$$

(6) $$\begin{array}{r} \phi_{\text{c}}=\phi_{\text{c}0},\;\phi_{\text{b}}=\phi_{\text{b}0},\;n=1,\;r=R\left(t\right). \end{array}$$

We assume a uniform initial tumor volume fraction ϕ_c0_ = 0.8 across the spheroid (Byrne and Preziosi, 2003) and, to model bacteria administration, we consider a time dependent value for the bacterial volume fraction at the spheroid outer radius:

(7) $$\begin{array}{r} \phi_{\text{b}}=\{\begin{array}{cc} 0, & \text{for}0\leq t<t_{0}\\ \phi_{\text{b}0}, & \text{for}t_{0}\leq t<t_{\text{a}}\\ 0, & \text{for}t_{\text{a}}\leq t\leq t_{\text{f}}, \end{array} \end{array}$$

where ϕ_b0_ is the administered volume fraction of bacteria, *t*_0_ is the time of administration and *t*_a_ its duration. Regarding the initial conditions, we consider a spheroid devoid of bacteria and displaying a uniform TC volume fraction and nutrient concentration over its radius:

(8) $$\begin{array}{r} \phi_{\text{c}}\left(r,0\right)=\phi_{\text{c}0},\;\phi_{\text{b}}=0,\;n=1. \end{array}$$

Finally, we prescribe an initial spheroid radius, i.e., *R*(0) = 90μm. The equations of the model are discretized through the Finite Element Method and solved using the commercial software COMSOL Multiphysics (COMSOL AB, 2019).

### 2.2. Choice of Mass Exchange Terms

**Figure 1:**
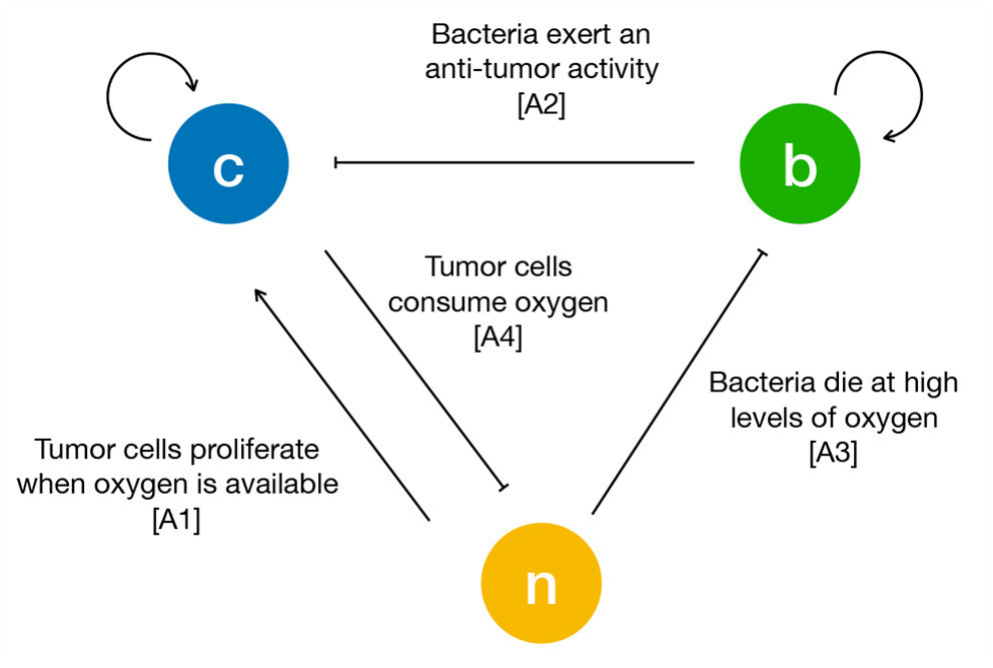
Schematic of the interactions between tumor cells (*c*), bacteria (*b*) and oxygen (*n*). The arrows are drawn according to the biological hypotheses detailed in the main text.

To formulate the mass exchange terms in Equations (1)–(3) we assume the following assumptions (see Figure 1):

**A1** TCs proliferate when oxygen is available. As soon as the latter decreases below a critical threshold, they stop proliferating and start necrosis (Chaplain et al., 2006; Gerlee and Anderson, 2007; Agosti et al., 2018).
**A2** Bacteria compete with TCs for space and exert an anti-tumor effect by a variety of mechanisms (e.g., by realizing toxins and therapeutic agents, or stimulating an immune response) (Forbes, 2010; Osswald et al., 2015; Torres et al., 2018; Zhou et al., 2018).
**A3** Bacteria die when oxygen is above a critical threshold and thrive in hypoxic conditions (*anaerobic* bacteria) (Toley and Forbes, 2011; Osswald et al., 2015; Phaiboun et al., 2015).
**A4** TCs consume oxygen provided by the culture medium (Matzavinos et al., 2009; Grimes et al., 2014).

The resulting mass exchange terms read:

(9) $$\begin{array}{r} S_{\text{c}}=\gamma_{\text{c}}\phi_{\text{c}}\frac{\phi_{\text{f}}}{\phi_{\text{f}0}}H\left(\frac{n}{n_{\text{cr}}}-1\right)-\delta_{\text{c}}\phi_{\text{c}}H\left(1-\frac{n}{n_{\text{cr}}}\right)-\kappa \phi_{\text{c}}\phi_{\text{b}}, \end{array}$$

(10) $$\begin{array}{r} S_{\text{b}}=\gamma_{\text{b}}\phi_{\text{b}}\frac{\phi_{\text{f}}}{\phi_{\text{f}0}}H\left(1-\frac{n}{n_{\text{cr}}}\right)-\delta_{\text{b}}\phi_{\text{b}}H\left(\frac{n}{n_{\text{cr}}}-1\right), \end{array}$$

(11) $$\begin{array}{r} S_{\text{n}}=-\delta_{\text{n}}\phi_{\text{c}}n. \end{array}$$

Here γ_i_ and δ_i_ are the proliferation and death rate of the i-th phase respectively (i = c, b), whereas δ_n_ is the oxygen consumption rate. In addition, ϕ_*f*0_ is the initial volume fraction of extracellular material and we indicate with H(·) a smooth version of the step function, and with *n*_cr_ the critical oxygen value below which hypoxic conditions develop. Finally, we do not consider a specific form for the anti-tumor effect of bacteria and introduce an effective TC killing rate κ in the equation for *S*_c_.

### 2.3. Model Parametrization

The parameters used in the model simulations are reported in Table 1. In the following we will compare model results with a set of published experiments on the U87 glioma cell line (Mascheroni et al., 2016). Colombo and colleagues (Colombo et al., 2015) estimated cell motility for glioma cells in the brain to be in the range [0.16, 0.5] mm^2^d^−1^. Since we deal with *in vitro* experiments where cells experience less constraints with respect to tissues, we assume the largest value in the range for the motility coefficient *D*_*c*_. We take the TC proliferation rate from the available data provided from the Bioresource Core Facility of the Physical Sciences-Oncology Center (PBCF, 2012), where the cell duplication time for U87 cells cultured *in vitro* is reported to be of 34 h. TC death rate in anoxic conditions has been estimated in Mart́ınez-González et al. (2012) to be on average of about 48 h. In their work, Toley and Forbes (2011) performed *in vitro* experiments for the migration of different bacteria strains inside tumor tissues. They estimated the motility of a Salmonella strain to be in the range [0.02, 0.05] mm^2^d^−1^ and its growth rate of about 6 h^−1^. Regarding bacteria death rate under starvation, Phaiboun et al. (2015) quantified bacterial death in a starving population of *E. coli*. They reported a mean value of 0.018 h^−1^, which we rounded up to 0.24 d^−1^ in our simulations. The oxygen diffusion coefficient has been quantified in several *in vitro* experiments and we assume a value for *D*_*n*_ of 100 mm^2^d^−1^, according to the estimates in Schaller and Meyer-Hermann (2005), Matzavinos et al. (2009), Grimes et al. (2014), Colombo et al. (2015), and Alfonso et al. (2016). For the oxygen consumption rate we make use of the arguments in Frieboes et al. (2009), where the authors estimated δ_*n*_ in spheroids from measuring the oxygen penetration length *L* and the diffusion coefficient *D*_*n*_ through the relation $\delta_{n}=D_{n}/L^{2}$. Since *L* was found in the order of 100μ*m*, they estimated $\delta_{n}=8,640\;{\text{d}}^{-1}$, a value that falls in the range reported in Alfonso et al. (2016) for gliomas. In the case of bacteria, two parameters were difficult to assess in tissues namely the chemotactic coefficient χ and the rate of anti-tumor activity. For the first, this is generally calculated for suspensions of bacteria that are stimulated from some kind of attractant. Ford and colleagues reported the chemotactic and random motility coefficients for bacteria solutions from different strains (Ford et al., 1991; Lewus and Ford, 2001). They provided values for Salmonella, which we used to estimate the range of chemotaxis in spheroids. We considered the value for the motility coefficient that they provided and divided it by the motility that we found for Salmonella in tissues (*D*_*b*_). Then, we scaled their chemotaxis coefficient with this value to obtain the chemotactic coefficient χ for bacteria in spheroids. For the bacteria anti-tumor activity rate, we were not able to find any estimate in the literature. Therefore, we varied κ over the range [0, 5] d^−1^, which includes processes that span multiple days to a few hours. Finally, we fitted the parameter for the critical oxygen concentration *n*_*cr*_ from the spheroid experiments in Mascheroni et al. (2016). The value that we found is similar to the one reported in Gerlee and Anderson (2007); Agosti et al. (2018) for tumor aggregates, which is in the range [0.15, 0.5].

**Table 1:** Summary of the parameter values considered in the model simulations.

**Parameter**	**Range and/or specific value**	**Description**	**References**
*D*_c_	[0.16, 0.5], 0.5mm^2^d^−1^	TC motility coefficient	Colombo et al., 2015
γ_c_	0.48d^−1^	TC proliferation rate	PBCF, 2012
δ_c_	0.5d^−1^	TC death rate	Mart́ınez-González et al., 2012
*D*_b_	[0.02, 0.05], 0.05mm^2^d^−1^	Bacterial motility coefficient	Toley and Forbes, 2011
γ_b_	15d^−1^	Bacterial proliferation rate	Gibson et al., 2018
δ_b_	0.24d^−1^	Bacterial death rate	Phaiboun et al., 2015
*D*_n_	100mm^2^d^−1^	Oxygen diffusion coefficient	Matzavinos et al., 2009
δ_n_	[0.46, 9.21] × 10^4^, 8640d^−1^	Oxygen consumption rate	Colombo et al., 2015
χ	[0, 0.864]mm^2^d^−1^	Bacterial chemotactic coefficient	estimated
κ	[0, 5]d^−1^	Bacterial killing rate	model specific
*n*_cr_	0.58	Critical oxygen concentration	calibrated

*The specific value used is listed after the range, when the latter is available*.

## 3. Results

### 3.1. Model Calibration on Spheroid Experiments

We start the analysis by considering the growth of a spheroid suspended in culture medium, in the absence of bacteria. We compare the results of the model with the data for radial growth of U87 tumor spheroids available from Mascheroni et al. (2016). We use the model to fit the critical oxygen concentration parameter *n*_cr_, keeping all the other quantities as defined in Table 1.

**Figure 2:**
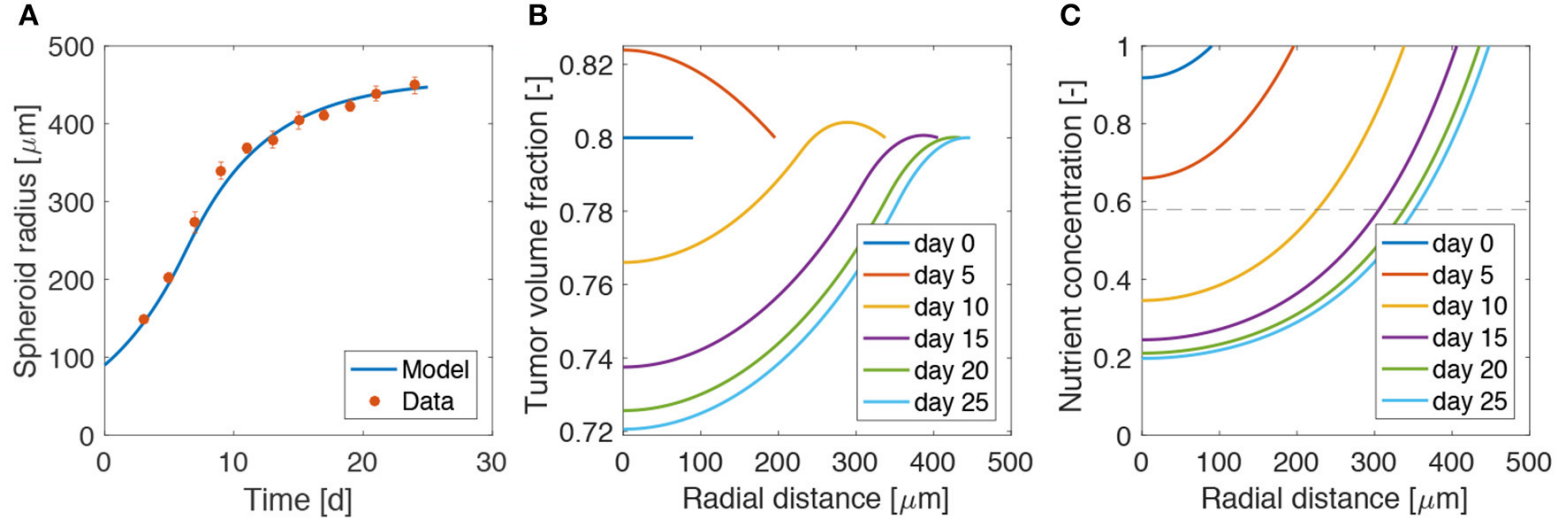
Calibration of the model on tumor spheroid data. **(A)** Fit of the mathematical model to the experimental data for the spheroid growth curve. The experimental points are taken from Mascheroni et al. (2016) and represent the growth of U87 spheroids. Dots are mean values and bars standard deviation of the measurements. Tumor volume fraction **(B)** and oxygen concentration **(C)** at different times of spheroid growth. The dashed line in the last plot displays the critical oxygen concentration.

Figure 2 shows a good agreement between the model and the experiments, over all the growth curve. The model is able to reproduce the two phases of spheroid growth usually described in the literature (Conger and Ziskin, 1983; Sutherland, 1988; Vinci et al., 2012). The spheroid radius (see Figure 2A) displays a first stage of rapid increase, followed by a saturation phase. This behavior is detailed in Figures 2B,C, showing the evolution of the tumor volume fraction and oxygen concentration over the spheroid radius at different time points. The tumor volume fraction, i.e., ϕ_c_, increases over the spheroid at early time points (Figure 2B). Then, as TCs consume oxygen to proliferate, its concentration decreases in the center of the aggregate (Figure 2C). When the oxygen level drops below the critical threshold *n*_cr_ (dashed line in Figure 2C), TCs stop proliferating and die. This results in a decrease of ϕ_c_ in the spheroid core, displayed at longer times in Figure 2B. Close to saturation, the amount of cells that proliferate is balanced by the number of cells that die, turning into extracellular material. Therefore, even if cell growth continues to take place in the outer rim of the spheroid, it is not enough to advance the spheroid front, which reaches a steady state. These results match qualitatively what is observed in the experimental (Landry et al., 1982; Montel et al., 2011; Grimes et al., 2014; Sarkar et al., 2018) and modeling (Ward and King, 1999; Byrne and Preziosi, 2003; Ambrosi and Mollica, 2004; Schaller and Meyer-Hermann, 2005; Mascheroni et al., 2016; Boemo and Byrne, 2019) literature for tumor spheroids and will serve as a basis for the discussion in the next sections.

### 3.2. Administration of Bacteria Leads to Tumor Remission but Not Eradication

**Figure 3:**
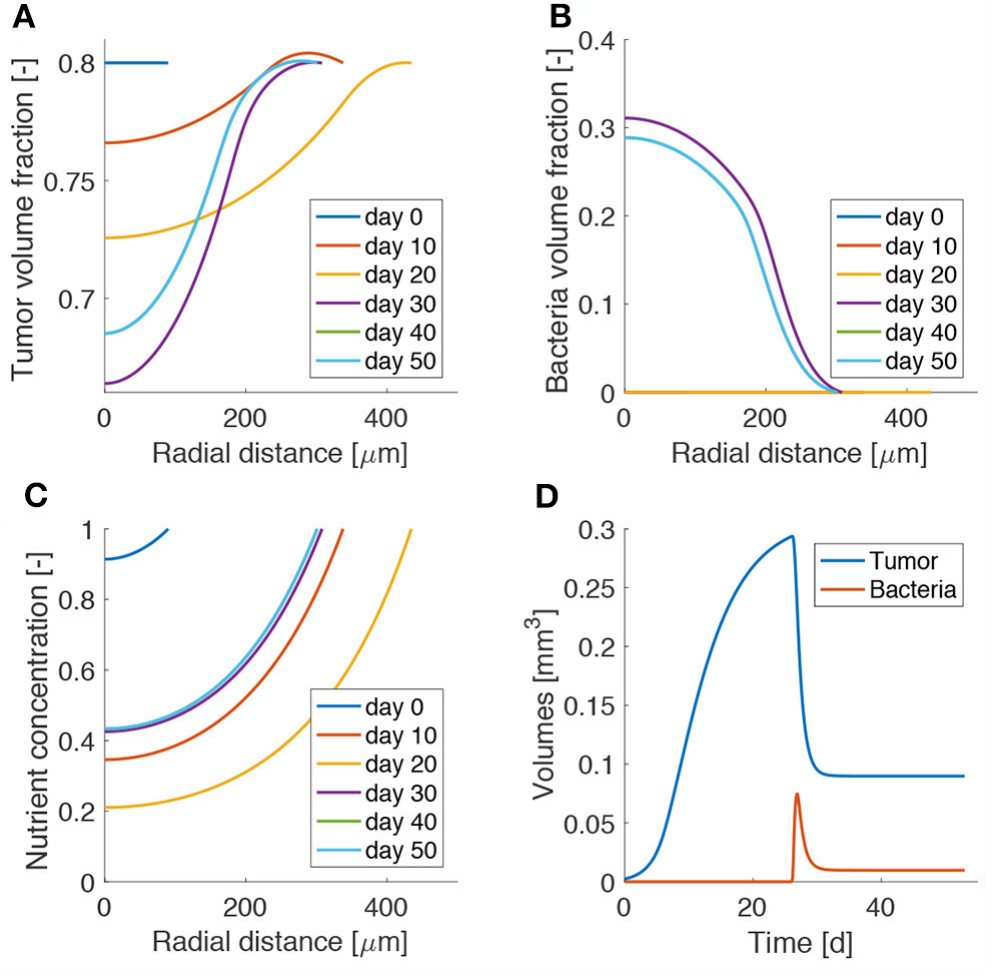
Model results for bacteria administration to tumor spheroids. Spatio-temporal evolution of tumor **(A)** and bacteria **(B)** volume fractions and oxygen concentration **(C)**. **(D)** Temporal evolution of tumor and bacteria volumes in the spheroid. The lines for day 40 are behind those for day 50, as the spheroid has reached a steady state.

Figure 3 shows the influence of bacterial therapy on tumor spheroid composition for an example case. We evaluate the effects of adding bacteria to the culture medium after the spheroid is fully formed, i.e., when hypoxic regions have developed. In particular, we select an administration time of *t*_0_ = 26d and a treatment duration of *t*_a_ = 2d. We consider an intermediate value for both the bacterial chemotactic coefficient and killing rate (χ = 0.432 mm^2^d^−1^, κ = 2.5 d^−1^). As shown by the low TC volume fraction in Figure 3A at later times, bacteria administration leads to spheroids less populated by TCs. This space is occupied by bacteria (Figure 3B), which thrive in the hypoxic region located in the spheroid core. After bacterial therapy the spheroid shrinks and is less populated by cancer cells. This leads to higher values of oxygen concentration at the center of the aggregate, as displayed in Figure 3C. Finally, Figure 3D shows the evolution of TC (*V*_c_) and bacteria (*V*_**b**_) volumes over time. These quantities are calculated as

(12) $$\begin{array}{r} V_{\text{i}}=\underset{V_{\text{sf}}}{\int }\phi_{\text{i}}dV, \end{array}$$

where the integral is performed over the spheroid volume *V*_sf_ (i = c,b). At early time points, *V*_c_ is in a phase of fast growth, since the nutrient is available throughout the spheroid and no bacteria are present. After administration, there is a fast increase of bacteria volume together with a rapid decrease of TC volume. At later time points the system evolves toward a steady state in which both bacteria and TCs coexist in the tumor aggregate. Even though the TCs are not completely removed, the spheroid persists in an equilibrium state, where an asymptotic size is kept for long times.

### 3.3. High Chemotaxis Allows for Maximal Reduction of Tumor Size

**Figure 4:**
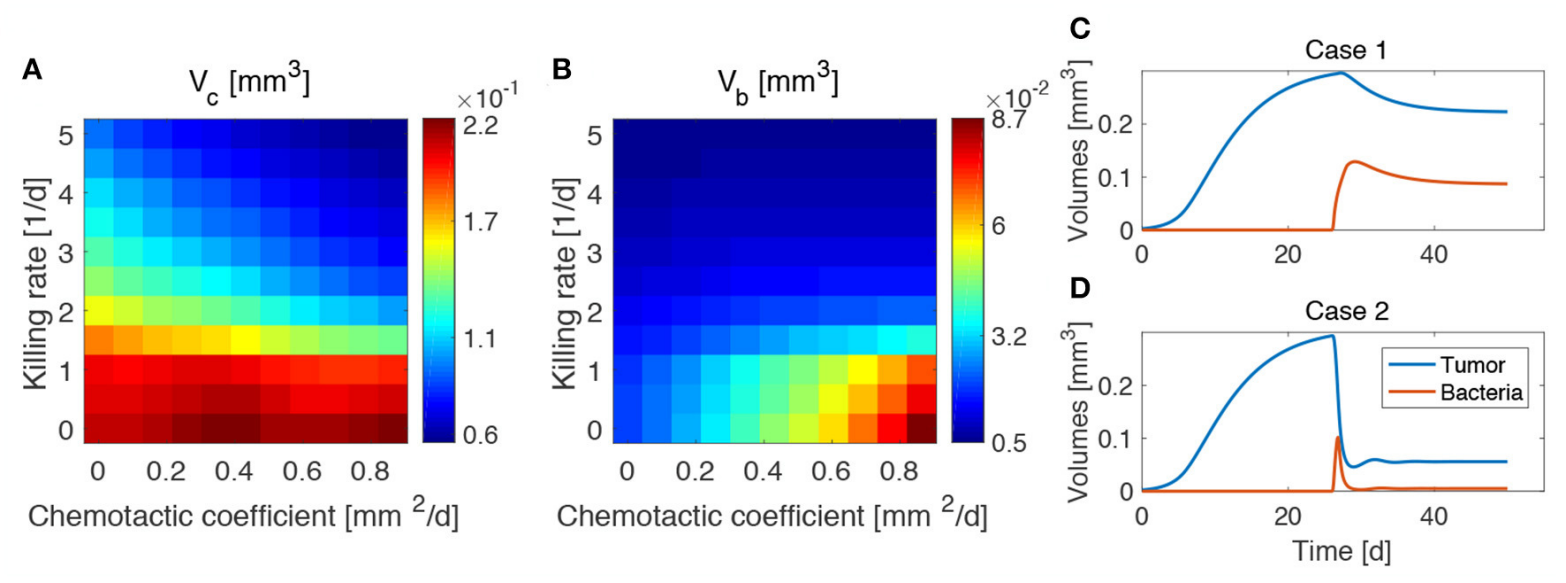
Influence of bacteria chemotactic coefficient and anti-tumor activity on tumor **(A)** and bacteria **(B)** volumes at the end of the simulations (day 50). Temporal evolution of tumor and bacteria volumes for a high chemotactic coefficient and a low (Case 1, **C**) and high (Case 2, **D**) killing rate.

We investigated the impact of different bacterial chemotactic and anti-tumor strengths on spheroid composition at the end of the simulations, i.e., at day 50 (Figure 4). We found that the highest reduction in tumor volume is obtained for the highest values of the chemotactic coefficient and killing rate, as shown in Figure 4A. On the other hand, highly chemotactic bacteria without an anti-tumor activity lead to the highest tumor volume. Interestingly, the tumor is never completely eradicated over all the explored parameter sequence. A similar result is obtained for the bacteria volume at the end of the simulations (Figure 4). Here, no matter the strength of chemotaxis or anti-tumor activity, bacterial cells are always present in the final spheroid volume. High bacterial volumes are present for high chemotactic coefficients, whereas high killing rates lead to small bacterial volumes independent of the chemotactic strength. Indeed, even though the tumor volume considerably varies over the chemotactic space for high killing rates, the bacterial volume is almost independent of this quantity (see Figure S1). Finally, Figures 4C,D show the temporal variation of tumor and bacterial volumes for two extreme cases occurring for high chemotaxis and low (Case 1) or high (Case 2) killing rate. The first plot shows that after the administration of bacteria the tumor volume is reduced, even in the absence of anti-tumor activity. The two populations in the spheroid reach an equilibrium at later times, with bacteria representing a significant portion of the spheroid. In the second case, the high anti-tumor activity of the bacteria is responsible for a sharp decrease of the tumor population, leading also to oscillations in the TC volume. Although bacteria now constitute a small part of the overall spheroid volume, they are still able to keep the tumor size under control.

### 3.4. Highly Proliferating and Low Oxygen Consuming Tumors Are Mostly Benefited From Bacterial Therapy

**Figure 5:**
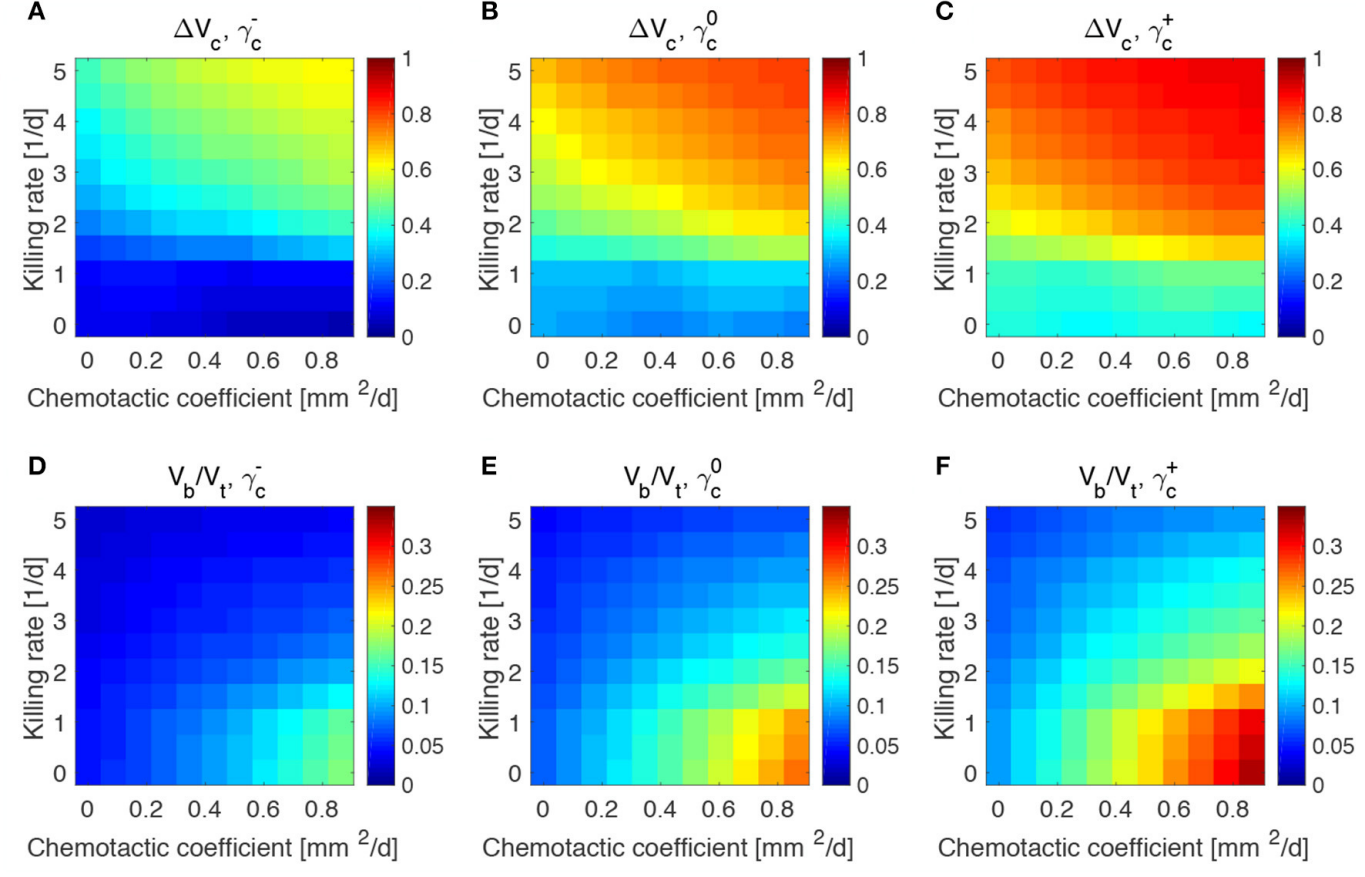
Impact of tumor proliferation rate on tumor and bacteria volumes at the end of the simulations (day 50). Relative tumor volume change and relative bacteria volume for low **(A,D)**, nominal **(B,E)** and high **(C,F)** tumor cell proliferation rate.

**Figure 6:**
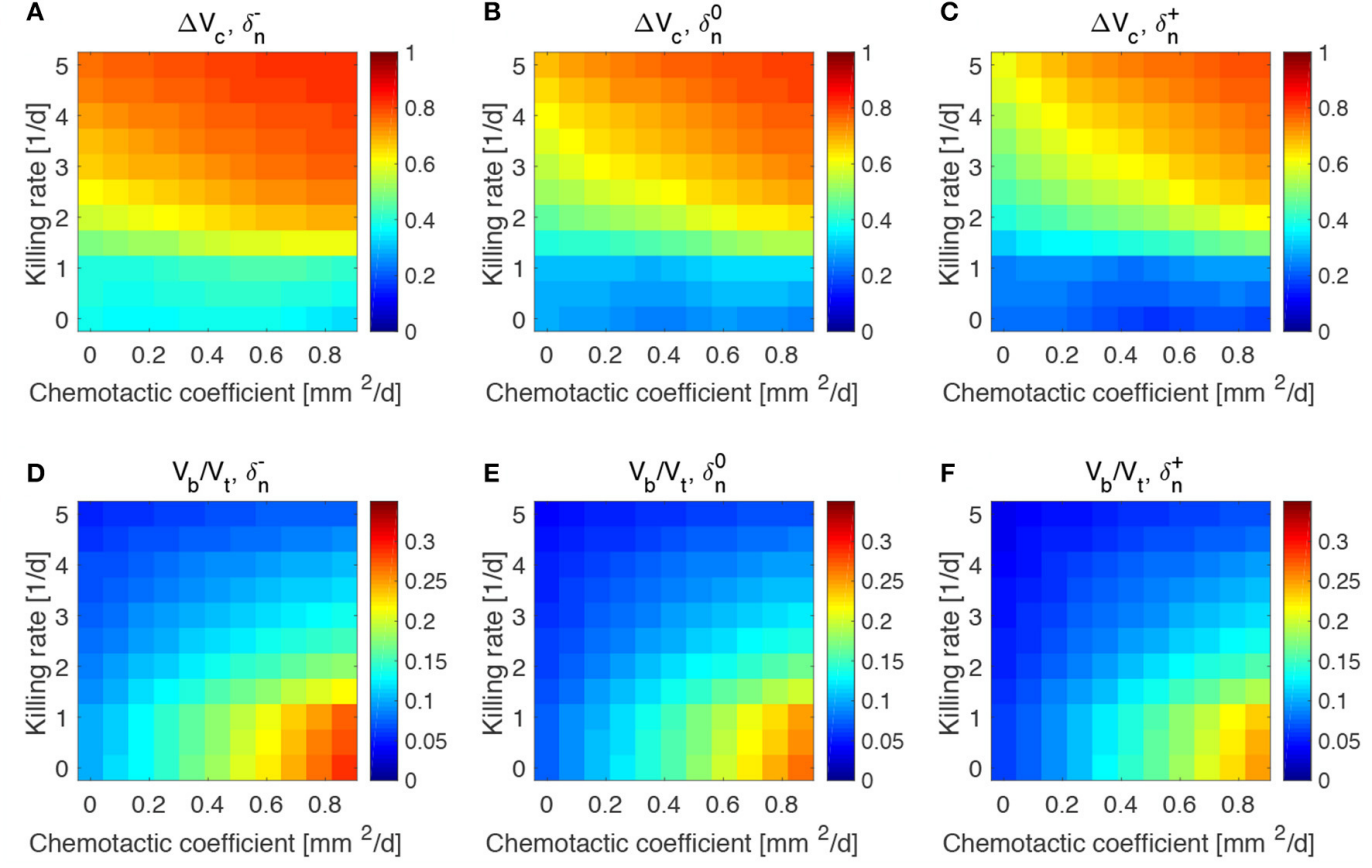
Impact of tumor oxygen consumption rate on tumor and bacteria volumes at the end of the simulations (day 50). Relative tumor volume change and relative bacteria volume for low **(A,D)**, nominal **(B,E)** and high **(C,F)** tumor cell proliferation rate.

The results obtained in the previous subsection are insensitive of the administration time *t*_0_, the duration of the administration *t*_a_ and the administered bacteria volume fraction ϕ_b0_, even for large variations of these parameters (see Figures S2–S4). This made us investigate whether the steady states reached at the end of the simulations and displayed in Figure 4 were therefore a function of the mechanisms regulating the tumor/bacteria dynamics. To check this hypothesis we simulated the behavior of TCs with a lower or higher proliferation and oxygen consumption rates with respect of the one shown in Figure 4. We report our findings in Figures 5, 6.

We considered a variation of ±50% with respect to the nominal value of the parameters in Table 1, and labeled the cases using the plus or minus in the superscript accordingly. All the other parameters keep the nominal values. We evaluated the spheroid response in terms of relative tumor reduction by introducing the quantity:

(13) $$\begin{array}{r} \Delta V_{\text{c}}=\frac{V_{0}-V_{\text{c}}}{V_{0}}, \end{array}$$

where *V*_c_ is the final tumor volume and *V*_0_ the tumor volume at the time of bacteria administration. We also analyzed the relative bacteria volume at the end of the simulation, plotting the ratio of bacteria volume *V*_b_ to the total spheroid volume *V*_t_.

Tumors in which cells proliferate at a higher rate display the highest tumor reductions (Figures 5A–C). This is particularly true for the treatment with bacteria characterized by high chemotaxis and killing rate. Highly proliferative tumors are the ones that also show higher colonization by bacteria, as displayed in Figures 5D–F. Treatments with high chemotactic bacteria with low killing rates provide the highest relative bacteria volumes. Low oxygen consumption by TCs leads to results similar to highly proliferative tumors (Figure 6). Again, treatment using bacteria with high chemotaxis and high killing rate produces the best results in terms of tumor reduction. Regarding the final bacterial content, both high and low oxygen consuming tumors show considerable bacteria colonization. As before, the relative bacteria volume is higher for highly chemotactic bacteria with low anti-tumor activity. Even though highly proliferative and low oxygen consuming TCs originate the highest final spheroid volumes (Figure S5), they benefit the most from bacteria treatment and display the higher final bacteria content.

## 4. Discussion

We proposed a mathematical model to study the influence of bacteria treatment on avascular tumor growth. We considered anaerobic bacteria which thrive in hypoxic environments and actively migrate toward nutrient deprived regions in solid tumors. The model was calibrated to reproduce published tumor spheroid data and then used to evaluate the impact of bacteria chemotaxis and killing rate on spheroid response.

Model results show preferential bacteria accumulation in the hypoxic spheroid core, with tumor cells more localized toward the external spheroid surface. In general, highly chemotactic bacteria possessing increased anti-tumor activity provide the highest tumor reduction after treatment. On the other hand, high chemotaxis but low anti-tumor activity lead to smaller tumor reduction but higher bacteria colonization at the end of the simulations. When varying the tumor parameters, we found that bacteria treatment works best for highly proliferative and low oxygen consuming tumors.

For simplicity, we considered a general effective anti-tumor activity of TCs by bacteria without focusing on specific mechanisms, e.g., cytotoxic agents, prodrug-converting enzymes, etc. (Kramer et al., 2018; Torres et al., 2018; Zhou et al., 2018). Such treatment modalities could be incorporated by extending the model, to provide a more accurate description of the therapeutic action. Moreover, we focused on tumor spheroids, an *in vitro* approximation of avascular tumors. As such, they lack all the interactions between the tumor and its immune environment. On the one hand, this approach allows to investigate the mutual dynamics of bacteria and tumor cells without external influences, but on the other including the cross-talk between bacteria and the components of the immune system would be a fundamental step to address questions coming from *in vivo* tumors. Following Boemo and Byrne (2019), we modeled the mechanical response of cells and bacteria in the simplest way considering the phases as inviscid fluids. Although this description is still able to qualitatively describe the experimental results, more detailed constitutive assumptions for the mechanical behavior of the phases would lead to new insights into the interactions between bacteria and TCs in the aggregate (Sciumè et al., 2013; Giverso et al., 2015; Ambrosi et al., 2017; Fraldi and Carotenuto, 2018; Mascheroni et al., 2018; Giverso and Preziosi, 2019). We also considered ideal spherical spheroids to reduce the mathematical problem to one dimension. Even if the qualitative results will be maintained in a three-dimensional geometry, adopting the latter will be crucial to translate the model to *in vivo* situations. Finally, a note should be made about model parametrization. Generally, mathematical models in the biological framework require several parameters to be determined (Alber et al., 2019) and this work is no exception. We listed the source of the parameters used in the simulations and performed a sensitivity analysis on tumor and bacterial parameters in Figures S6, S7, but a thorough analysis on this topic has been not carried out for the sake of brevity. We believe that a dedicated study on this aspect, particularly in terms of the “Sloppy” model framework (White et al., 2016), could be extremely interesting for this model and for all the biomedical mixture model class.

In this modeling approach, space competition between bacteria and tumor cells arises naturally from the conservation of mass and momentum imposed by the governing equations. As no void regions are allowed into the spheroid, when cells move or die one of the model components automatically fills the space. Bacteria and TCs compete for space in the spheroid and the expansion of the tumor becomes limited, especially when the anti-tumor activity of bacteria is strong. However, for increasing values of the chemotactic coefficient and low values of the killing rate, bacteria localize predominantly in the spheroid core and displace TCs to the outer region of the spheroid. Both types of cell can proliferate in each of the two spheroid areas (hypoxic for spheroids, well-oxygenated for TCs), giving rise to high spheroid volumes and considerable bacteria colonization.

As a matter of fact, chemotaxis could be a target for bacteria-based anticancer therapies and diagnostic tools. For example, TCs that become restricted to outer spheroid areas after administration of highly chemotactic bacteria are more oxygenated and could benefit from standard chemotherapeutic or radiation treatments in the context of synergistic treatments (Zhou et al., 2018). We highlight that this is an example showing that mathematical models could help to identify situations when TC sensitization to therapies might be possible - see also (Owen et al., 2004; Kim et al., 2013; Michor and Beal, 2015; Mascheroni et al., 2017). On the other hand, highly chemotactic bacteria could be used as tracers to identify necrotic regions in spheroid, exploiting their targeting efficiency. Moreover, the simulations show the existence of steady states in which a small population of bacteria is in dynamical equilibrium with cancer cells, leading to tumor size control over time. All these mechanisms arise as a pure physical effect from the competition for space between cancer and bacteria cells and could be optimized to obtain the highest tumor volume reduction or bacteria colonization. Currently, even though researchers are aware of the benefits coming from active bacteria migration toward hypoxic regions in tumors (Forbes, 2010; Kramer et al., 2018), this knowledge has not been efficiently exploited in the clinical trials carried out so far (Torres et al., 2018).

Finally, we point out a few straightforward developments that emerge from the findings of this work. Firstly, our theoretical results advocate for experiments with tumor spheroids. With such a simplified experimental setup, several bacterial strains could be tested on different cancer cell lines to validate model findings. Secondly, the model could be extended to consider different synergistic treatments combined to bacterial administration. Chemotherapy or radiation therapy could be easily included in the model framework, exploiting the modular nature of the equations. The action of the immune system (in a co-culture in spheroids or for an *in vivo* situation) could be also integrated in future model versions. Lastly, we believe that the tight coupling between the dynamics of TCs and bacteria in terms of regulating their reciprocal environment could be suitably addressed via mathematical models, in order to control the bacterial infection or identify the optimal timing of the therapy.

## Data Availability Statement

The datasets generated for this study are available on request to the corresponding author.

## Author's Note

This manuscript has been released as a pre-print at BioRxiv (Mascheroni et al., 2020).

## Author Contributions

PM, HH, and MM-H contributed conception and design of the study. PM derived the model and performed the computational analysis. PM wrote the first draft of the manuscript. HH and MM-H wrote sections of the manuscript. All authors contributed to manuscript revision, read, and approved the submitted version.

## Conflict of Interest

The authors declare that the research was conducted in the absence of any commercial or financial relationships that could be construed as a potential conflict of interest.
