# Supplementary material for: Investigating the Physical Effects in Bacterial Therapies for Avascular Tumors
Source: Front Microbiol. 2020 Jun 4;11:1083. doi: 10.3389/fmicb.2020.01083 (PMC7287150; doi:10.3389/fmicb.2020.01083)
Supplement: Supplementary file 1 [file Data_Sheet_1.PDF]

Supplementary Information for: Investigating physical effects in bacterial therapies for avascular tumors

Pietro Mascheroni[a], Michael Meyer-Hermann[a,b,c,*], Haralampos Hatzikirou[a,*]

[a] *Braunschweig Integrated Centre of Systems Biology and Helmholtz Centre for Infection Research, Braunschweig, Germany.*
[b] *Centre for Individualized Infection Medicine, Hannover, Germany.*
[c] *Institute for Biochemistry, Biotechnology and Bioinformatics, Technische Universität Braunschweig.*

1. Model derivation

Model derivation is carried out in the framework of mixture theory, following the approach discussed in Preziosi (2003); Byrne (2012). Specifically, we adapt the derivation in Boemo and Byrne (2019), which deals with a mixture model for macrophage-based therapies in tumor spheroids, to our problem. We consider a mixture constituted by cancer cells (c), bacteria (b) and extracellular material (f) - *phases*, in the following. We also include the presence of a nutrient (n), i.e. oxygen, in our description. Following (Preziosi, 2003; Byrne, 2012), we first write the balance of mass for each phase:

$$\partial_t \phi_\mathrm{i} + \mathrm{div}\,(\phi_\mathrm{i} \boldsymbol{v}_\mathrm{i}) = S_\mathrm{i}, \tag{1}$$

in which ϕ_i, $\boldsymbol{v}_\mathrm{i}$ and S_i are the volume fraction, velocity and mass exchange term related to the i-th phase (i = c, b, f). Note that Equation (1) implicitly assumes that the phases have the same constant mass density. In the following we will also assume that the mixture is closed with respect to mass, so that mass can only be converted from one phase to the other, i.e. $S_\mathrm{f} = -S_\mathrm{c} - S_\mathrm{b}$.

*Correspondence:
mmh@theoretical-biology.de (M.M.H.);
haralampos.hatzikirou@helmholtz-hzi.de (H.H.)

In mixture theory velocity fields are determined by considering the mechanical response of the phases to mutual interactions Ambrosi and Preziosi (2002). Neglecting inertial effects, as usually done for growth phenomena (Preziosi, 2003; Byrne, 2012), the balance of linear momentum can be written as:

$$\mathrm{div}\,(\phi_{\mathrm{i}}\boldsymbol{\sigma}_{\mathrm{i}}) + \sum_{\mathrm{i}\neq\mathrm{j}} \boldsymbol{m}_{\mathrm{ij}} + p\,\mathrm{grad}\,(\phi_{\mathrm{i}}) = \boldsymbol{m}_{\mathrm{i}}. \tag{2}$$

Here $\boldsymbol{\sigma}_{\mathrm{i}}$ is the stress tensor of the i-th phase, $\boldsymbol{m}_{\mathrm{ij}}$ represent the forces exerted on the i-th phase by the j-th phase, and $\boldsymbol{m}_{\mathrm{i}}$ describes an external force acting on the i-th phase (i, j = c, b, f). Note that, for the action-reaction principle, $\boldsymbol{m}_{\mathrm{ij}} = -\boldsymbol{m}_{\mathrm{ji}}$. Finally, the terms $p\,\mathrm{grad}\,(\phi_{\mathrm{i}})$ represent interfacial effects between phases, with p being the interfacial pressure (Byrne, 2012). In this modeling framework, p emerges as a Lagrange multiplier due to the saturation constraint

$$\sum_{\mathrm{i}=\mathrm{c,b,f}} \phi_{\mathrm{i}} = 1, \tag{3}$$

meaning that we assume that there are no empty spaces within the mixture (Preziosi, 2003; Byrne, 2012).

We conclude the set of governing laws by stating an equation for the normalized nutrient concentration n in the mixture, i.e. the tumor:

$$\partial_t n = D_{\mathrm{n}}\mathrm{div}\,(\mathrm{grad}\,n) + S_{\mathrm{n}}, \tag{4}$$

in which D_{n} is the nutrient diffusion coefficient and S_{n} represents the nutrient mass exchange with the model phases.

1.1. Constitutive relationships

We close the model by selecting suitable constitutive assumptions. First, we assume that the interaction terms $\boldsymbol{m}_{\mathrm{ij}}$ depend linearly on the relative phase velocities (Preziosi, 2003; Byrne, 2012):

$$\boldsymbol{m}_{\mathrm{ij}} = -\mu\phi_{\mathrm{i}}\phi_{\mathrm{j}}\,(\boldsymbol{v}_{\mathrm{i}} - \boldsymbol{v}_{\mathrm{j}})\,, \tag{5}$$

with the same linearity constant μ for all the phases (i = c, b, f). We consider only a single external force $\boldsymbol{m}_{\mathrm{b}}$ acting on bacteria. This term describes bacteria chemotaxis following spatial hypoxic gradients and models

active cell migration towards waste products from dying cancer cells (Forbes, 2010; Toley and Forbes, 2011). We assume a linear relationship,

$$\boldsymbol{m}_{\mathrm{b}} = \phi_{\mathrm{b}} \chi_{\mathrm{b}} \operatorname{grad} n, \tag{6}$$

in which χ_{b} describes the strength of chemoattraction.

Following Breward et al. (2001, 2002); Byrne (2012); Boemo and Byrne (2019) we consider the phases as inviscid fluids and associate an interfacial pressure to each of them. For simplicity, we take the pressure in the extra-cellular material to be equal to that in the fluid surrounding the spheroid, p. The stress tensors in Equation (2) are defined such that the interfacial pressure of each phase is given by the pressure in the extracellular material plus a correction term, specific to its phase (Boemo and Byrne, 2019):

$$\boldsymbol{\sigma}_{\mathrm{f}} = -p\boldsymbol{I}, \tag{7}$$

$$\boldsymbol{\sigma}_{\mathrm{b}} = -(p + \pi_{\mathrm{b}})\boldsymbol{I}, \tag{8}$$

$$\boldsymbol{\sigma}_{\mathrm{c}} = -(p + \pi_{\mathrm{c}})\boldsymbol{I}, \tag{9}$$

where $\boldsymbol{I}$ is the identity tensor. The ratio π_{i}/μ characterizes the movement of the i-th phase in the mixture and is generally identified as the phase motility coefficient D_{i} (i = c, b) (Boemo and Byrne, 2019). In the following we will also define $\chi = \chi_{\mathrm{b}}/\mu$ as the bacterial chemotactic coefficient.

1.2. Spherical symmetry, initial and boundary conditions

After recasting Equation (1) in spherical symmetry and after imposing the saturation constraint in Equation (3), we have:

$$v_{\mathrm{c}}\phi_{\mathrm{c}} + v_{\mathrm{b}}\phi_{\mathrm{b}} + v_{\mathrm{f}}\phi_{\mathrm{f}} = 0, \tag{10}$$

in which v_{i} is the radial velocity of the i-th phase (i = c, b, f). Substituting Equations (5), (7)-(9) and (10) in Equation (2) we obtain for the radial velocities:

$$v_{\mathrm{c}} = D_{\mathrm{b}} \frac{\partial \phi_{\mathrm{b}}}{\partial r} + D_{\mathrm{c}} \left(1 - \frac{1}{\phi_{\mathrm{c}}}\right) \frac{\partial \phi_{\mathrm{c}}}{\partial r} + \chi \phi_{\mathrm{b}} \frac{\partial n}{\partial r}, \tag{11}$$

$$v_{\mathrm{b}} = D_{\mathrm{b}} \left(1 - \frac{1}{\phi_{\mathrm{b}}}\right) \frac{\partial \phi_{\mathrm{b}}}{\partial r} + D_{\mathrm{c}} \frac{\partial \phi_{\mathrm{c}}}{\partial r} - \chi \left(1 - \phi_{\mathrm{b}}\right) \frac{\partial n}{\partial r}, \tag{12}$$

after summing over the phases in Equation (2) to express p as a function of the other model quantities. Substituting Equations (11)-(12) in (1) and rewriting the system in spherical symmetry leads to the final model equations:

$$\frac{\partial \phi_\mathrm{c}}{\partial t} = \frac{1}{r^2}\frac{\partial}{\partial r}\left\{r^2\left[D_\mathrm{c}\left(1-\phi_\mathrm{c}\right)\frac{\partial \phi_\mathrm{c}}{\partial r} - D_\mathrm{b}\phi_\mathrm{c}\frac{\partial \phi_\mathrm{b}}{\partial r} - \chi\phi_\mathrm{c}\phi_\mathrm{b}\frac{\partial n}{\partial r}\right]\right\} + S_\mathrm{c}, \tag{13}$$

$$\frac{\partial \phi_\mathrm{b}}{\partial t} = \frac{1}{r^2}\frac{\partial}{\partial r}\left\{r^2\left[D_\mathrm{b}\left(1-\phi_\mathrm{b}\right)\frac{\partial \phi_\mathrm{b}}{\partial r} - D_\mathrm{c}\phi_\mathrm{b}\frac{\partial \phi_\mathrm{c}}{\partial r} + \chi\phi_\mathrm{b}\left(1-\phi_\mathrm{b}\right)\frac{\partial n}{\partial r}\right]\right\} + S_\mathrm{b}, \tag{14}$$

$$\frac{\partial n}{\partial t} = \frac{1}{r^2}\frac{\partial}{\partial r}\left(r^2 D_\mathrm{n}\frac{\partial n}{\partial r}\right) + S_\mathrm{n}. \tag{15}$$

Note that we do not solve for ϕ_f since it can be obtained as $\phi_\mathrm{f} = 1-\phi_\mathrm{c}-\phi_\mathrm{b}$ through Equation (3).

References

Ambrosi, D., Preziosi, L., 2002. On the closure of mass balance models for tumor growth. Mathematical Models and Methods in Applied Sciences 12, 737–754.

Boemo, M.A., Byrne, H.M., 2019. Mathematical modelling of a hypoxia-regulated oncolytic virus delivered by tumour-associated macrophages. Journal of Theoretical Biology 461, 102–116.

Breward, C., Byrne, H., Lewis, C., 2001. Modelling the interactions between tumour cells and a blood vessel in a microenvironment within a vascular tumour. European Journal of Applied Mathematics 12, 529–556.

Breward, C., Byrne, H., Lewis, C., 2002. The role of cell-cell interactions in a two-phase model for avascular tumour growth. Journal of Mathematical Biology 45, 125–152.

Byrne, H., 2012. Mathematics and life sciences.

Forbes, N.S., 2010. Engineering the perfect (bacterial) cancer therapy. Nature Reviews Cancer 10, 785.

Preziosi, L., 2003. Cancer modelling and simulation. CRC Press.

Toley, B.J., Forbes, N.S., 2011. Motility is critical for effective distribution and accumulation of bacteria in tumor tissue. Integrative Biology 4, 165–176.

2. Supplementary Figures

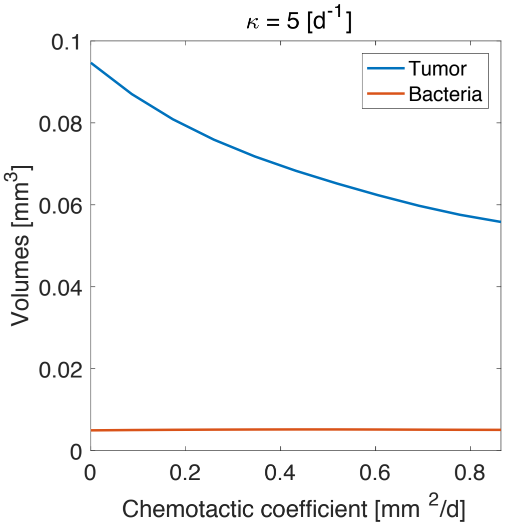

Figure S1: Influence of bacteria chemotactic coefficient on tumor and bacteria volume for high anti-tumor activity.

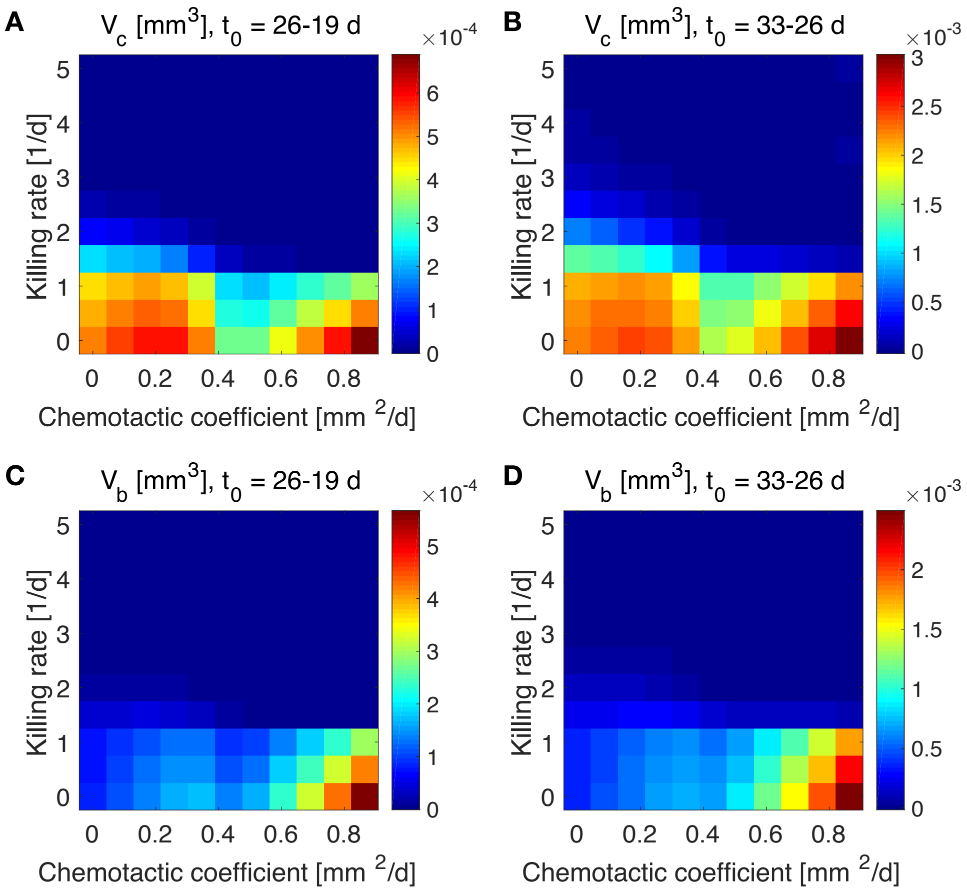

Figure S2: Influence of bacteria administration time t_0 on tumor and bacteria volumes at the end of the simulations (day 50). The maps show the difference between the volumes obtained using the parameters listed in the figure titles.

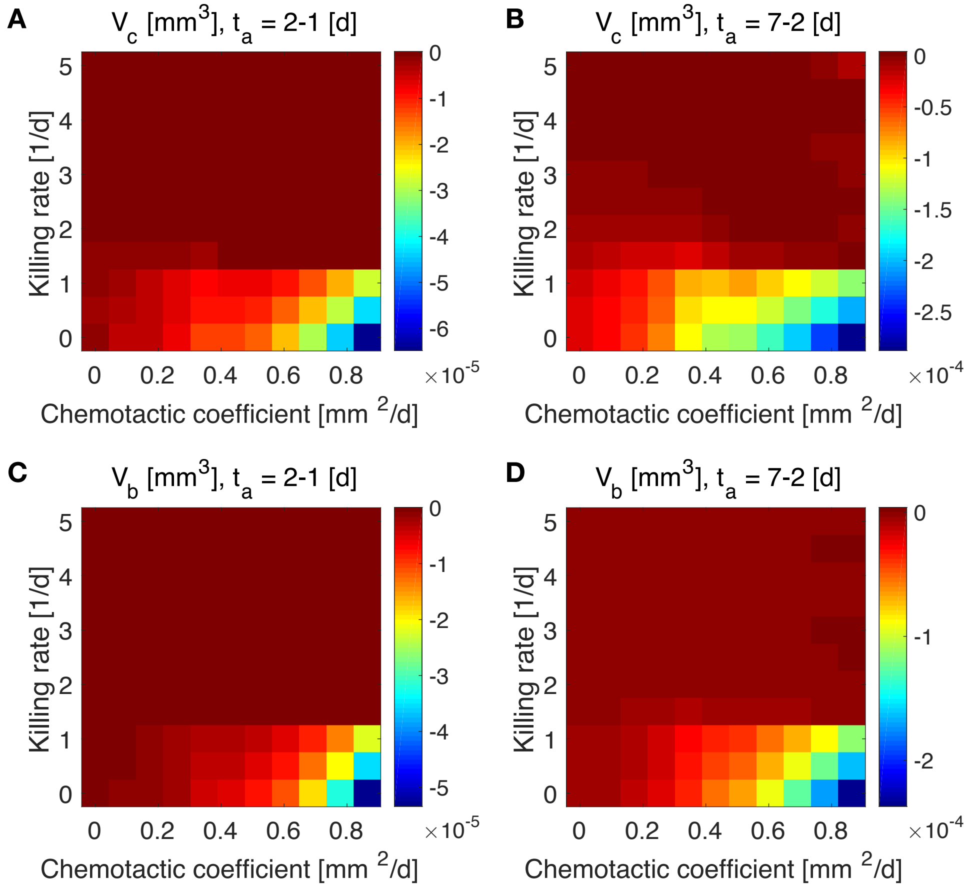

Figure S3: Influence of bacteria administration duration t_a on tumor and bacteria volumes at the end of the simulations (day 50). The maps show the difference between the volumes obtained using the parameters listed in the figure titles.

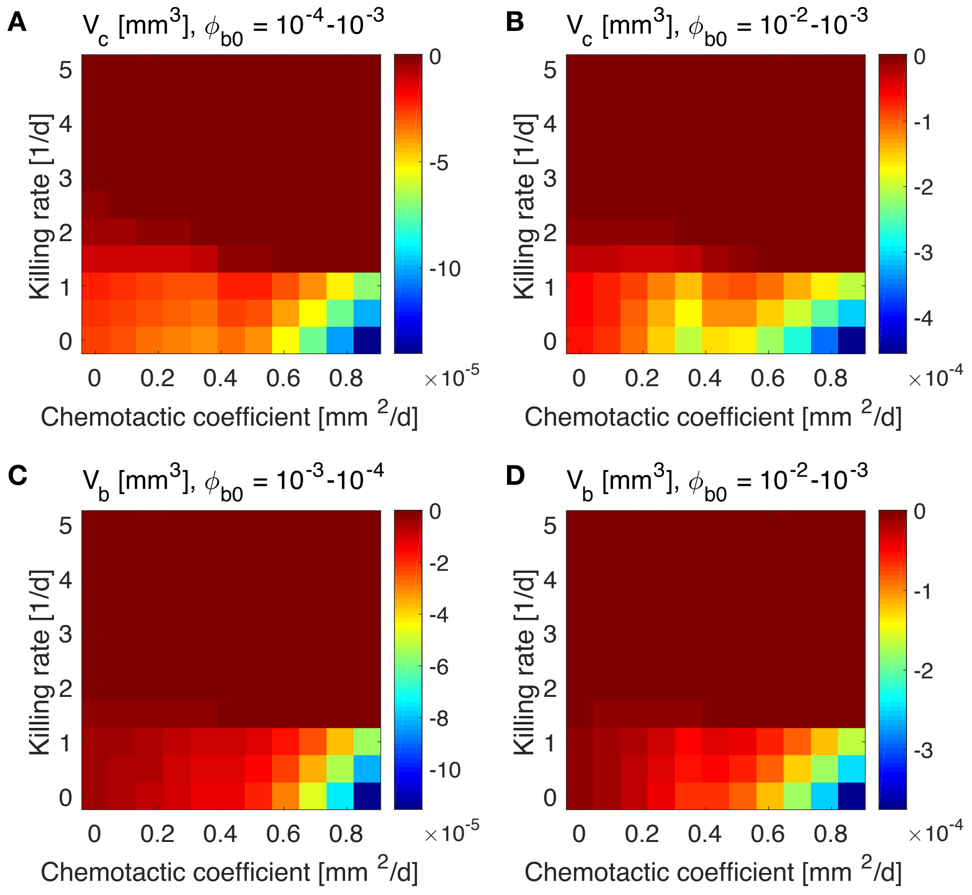

Figure S4: Influence of administered bacteria volume fraction ϕ_{b0} on tumor and bacteria volumes at the end of the simulations (day 50). The maps show the difference between the volumes obtained using the parameters listed in the figure titles.

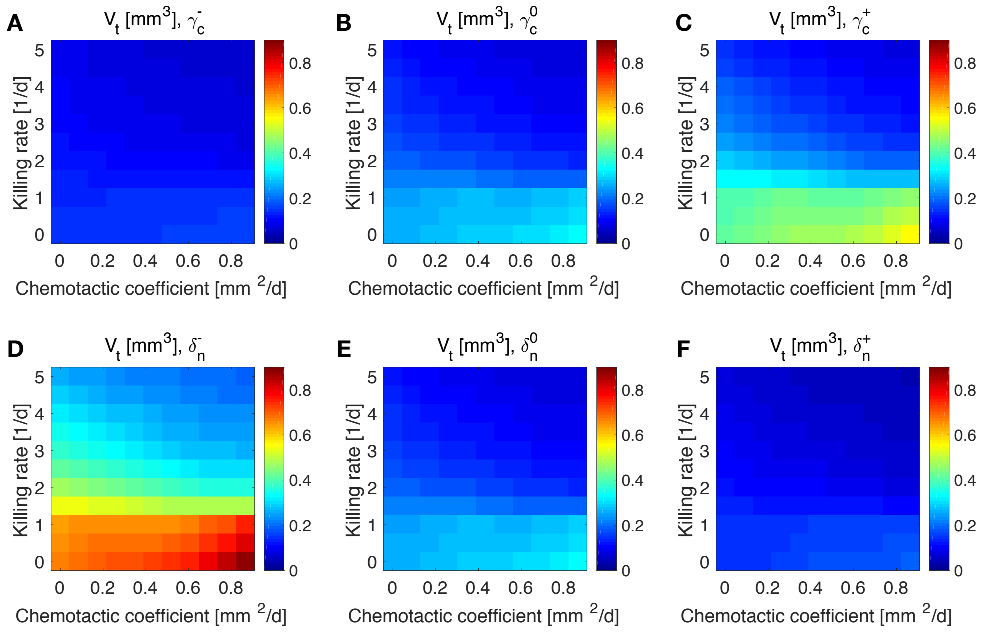

Figure S5: Final total spheroid volume for different parameters characterizing tumor cells. **A-C** Variation of tumor cell proliferation rate. **D-F** Variation of tumor cell oxygen consumption.

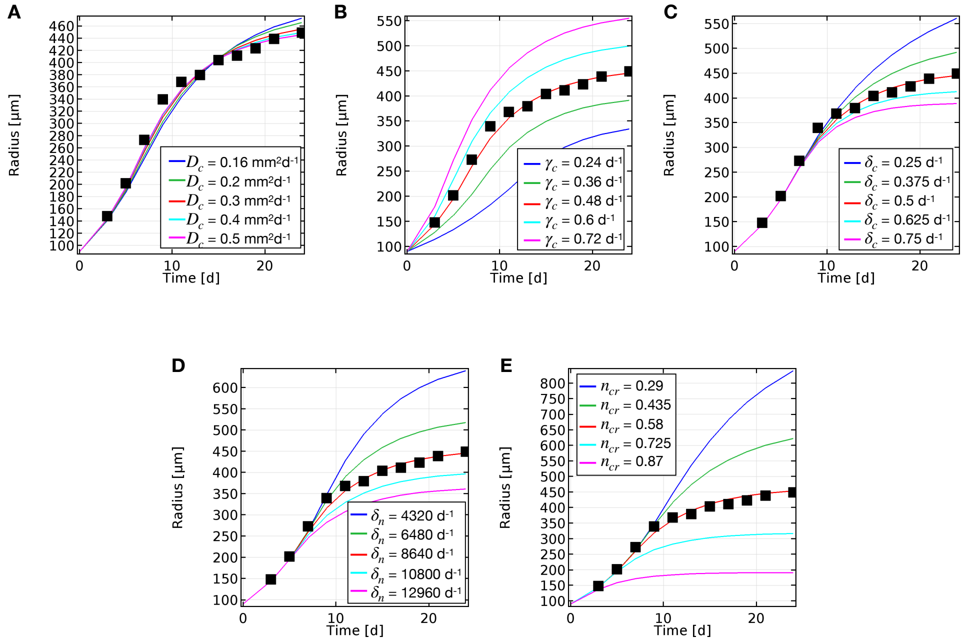

Figure S6: Sensitivity analysis for the growth of a spheroid in the absence of bacteria. In particualr, we show the influence of tumor cell motility (**A**), proliferation rate (**B**), death rate (**C**), oxygen consumption rate (**D**) and critical oxygen level (**E**) on spheroid radius.

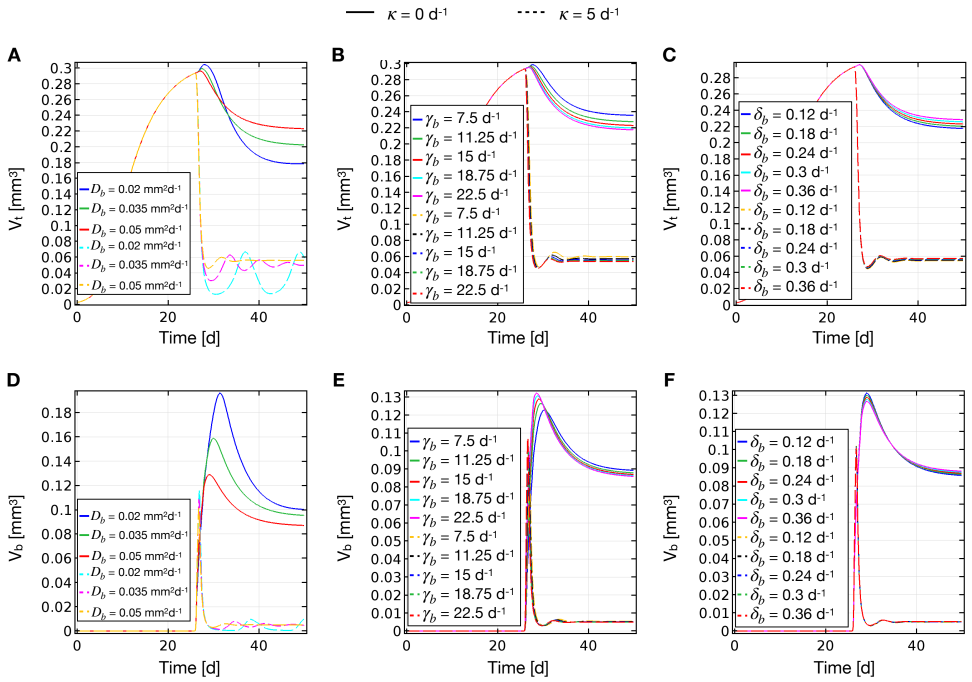

Figure S7: Sensitivity analysis for the growth of a tumor spheroid subjected to bacterial therapy. We analyzed the cases in which the chemotaxis coefficient was fixed to $0.864\text{mm}^2\text{d}^{-1}$ and the killing rate was set to 0 (solid lines) and 5d^{-1} (dashed lines). Tumor (**A**-**C**) and bacterial (**D**-**F**) volumes are plotted over time. We show the influence of bacterial motility (**A**,**D**), proliferation rate (**B**,**E**), and death rate (**C**,**F**) on both volumes over time.
